# Overdiagnosis of breast cancer in Denmark

**DOI:** 10.1038/sj.bjc.6601738

**Published:** 2004-03-16

**Authors:** P-H Zahl

**Affiliations:** 1 Norwegian Institute of Public Health, PO Box 4404, N-0403 Oslo, Norway

**Sir**,

Olsen *et al* (2003) claim in their article that organised mammography screening can operate without overdiagnosis of breast cancer.

This paper contains no statistical analysis and does not report the number of detected cancers, so the readers can do the proper statistical analysis themselves. In particular, it does not report the incidence increase in per cent, and the readers are left to look at some figures.

When I look at Figure 2, I read that the cumulative risk in Copenhagen increases from about 5.25% (1989–91) to 6.25% (1993–95). This gives 19% increase. Comparing the 5.75% cumulative risk on Fyn in 1992–93 to 6.75% cumulative risk in 1996–97 shows a 17% increase.

There was some opportunistic screening before organised screening started in Denmark (Olsen *et al*, 2003), so there is reason to attribute some of the incidence increase before 1993 to mammography. If cumulative risk in the second screening round is compared to the cumulative risk in 1987–89 (Copenhagen) or 1988–89 (Fyn), I get at least 30 and 50% incidence increases, respectively.

The authors should also have studied incidence in the age group 70–74 years after some years with screening in the age group 50–69 years (Spix *et al*, 2003). If mammography screening brings the time of diagnosis forward, incidence in the age group 70–74 years should decline. The difference between incidence increase in the age group 50–69 years and decline in the age group 70–74 years is the correct definition of overdiagnosis (Spix *et al*, 2003).

The authors do not report ductal carcinoma *in situ* (DCIS). On including DCIS (as Olsen and Gøtzsche (2001) did), one would assume that overdiagnosis today must be a much larger problem than reported by Olsen and Gøtzsche in the screening trials 20 years ago. In contrast, Etzioni *et al* (2002) recently reported only 30% overdiagnosis when screening with prostate-specific antigen for prostate cancer.

I think that this paper actually shows that overdiagnosis is a serious problem when screening with mammography.

## References

[bib1] Etzioni R, Penson DF, Legler JM, di Tommaso D, Boer R, Gann PH, Feuer EJ (2002) Overdiagnosis due to prostate-specific antigen screening: lessons from U.S. prostate cancer incidence trends. J Natl Cancer Inst 94: 981–990 12096083 10.1093/jnci/94.13.981

[bib2] Olsen O, Gøtzsche PC (2001) Cochrane review on screening for breast cancer with mammography. Lancet 358: 1340–1342 11684218 10.1016/S0140-6736(01)06449-2

[bib3] Olsen AH, Jensen A, Njor SH, Villadsen E, Schwartz W, Vejborg I, Lynge E (2003) Breast cancer incidence after the start of mammography screening in Denmark. Br J Cancer 83: 362–365 10.1038/sj.bjc.6600712 PMC2747539 12569377

[bib4] Spix C, Michaelis J, Berthold F, Erttmann R, Sander J, Schilling FH (2003) Lead-time and overdiagnosis in estimation in neuroblastoma screening. Stat Med 22: 2877–2892 12953286 10.1002/sim.1533

